# Unraveling the crosstalk among ethylene, nitric oxide, and polyamines in tailoring the abiotic stress resilience in plants

**DOI:** 10.1007/s44154-024-00198-2

**Published:** 2025-03-10

**Authors:** Arun Kumar Maurya, Rachna Agarwal, Ravi Gupta

**Affiliations:** 1 Multanimal Modi College, Modinagar, Ghaziabad, 201204 India; 2 Bhabha Atomic Research Centre, Trombay, Mumbai 400085 India; 3 Homi Bhabha National Institute, Anushaktinagar, Mumbai 400094 India; 4 Plant Stress Physiology and Proteomics Laboratory, College of General Education, Kookmin University, Seoul, 02707 South Korea

**Keywords:** Crosstalk, Ethylene, Nitric oxide, Phytohormones, Plant growth regulators, Polyamines, Signaling, Stress resilience

## Abstract

Abiotic stresses are the major factors affecting the growth and productivity of plants. After perceiving the stress, plants orchestrate sophisticated signaling to maximize their fitness under stress conditions which involves the synthesis or inhibition of various plant growth regulators (PGRs). Among others, ethylene (ET), polyamines (PAs), and nitric oxide (NO) are emerging as crucial PGRs that shape plant responses to various stresses. Interestingly, their biosynthesis is interconnected through common precursors, S-adenosyl methionine (SAM) and L-arginine; therefore, the generation of one affects the synthesis and signaling of the other. Oxidative stress, driven by the production of reactive oxygen species (ROS), is a common feature across all types of stress which triggers several downstream responses such as membrane damage and osmotic imbalance. The troika of ET-PA-NO works in harmony to ensure the maintenance of ROS homeostasis by activating enzymatic and nonenzymatic antioxidants, phytohormones and other PGRs, and several stress-related proteins. Moreover, this trio also tailors various stress-specific responses such as closing stomata under drought and UV-B stress, inducing anaerobic genes during hypoxia, limiting heavy metals uptake by modifying Casparian strip, and maintaining ion/osmotic homeostasis and membrane integrity during salinity and cold stress. In the present review, efforts have been made to present the interconnections among ET-PA-NO as well as their crosstalk in discrete abiotic stresses to unveil and understand their interrelated regulatory mechanisms.

## Introduction

Plants are exposed to fluctuating environmental conditions that tend to limit their growth and productivity. These conditions not only induce abiotic stresses but also create conducive conditions for biotic stress (Son and Park 2022). Plants perceive these changes through receptors and initiate signaling cascades that result in morpho-physio-biochemical and molecular alterations which facilitate adaptations to the altered environment. These adaptive responses include the synthesis of specialized signaling molecules such as phytohormones and plant growth regulators (PGRs) which perform various regulatory and protective roles (Gupta 2023). Phytohormones, the naturally produced signaling molecules, are known to regulate the growth and development in plants at very low concentrations, both during normal as well as challenging environmental conditions (Waadt et al. 2022). The classical five phytohormones include auxin (IAA), gibberellin (GA), cytokinin (CK), ethylene (ET), and abscisic acid (ABA) that are known to regulate plant processes right from embryogenesis to senescence and provide defense against diverse stresses (Ali et al. 2024; Waadt et al. 2022). Besides, several other molecules have been identified in recent decades with plant growth regulatory properties. These include nitric oxide (NO), brassinosteroids (BR), polyamines (PAs), melatonin, hydrogen sulfide (H_2_S), and strigolactones, among various others (Zahid et al. 2023). Intriguingly, many of these phytohormones and PGRs are interconnected, and often their crosstalk governs the stress response (Waadt et al. 2022). In particular, the interconnection among gaseous molecules ET and NO along with PAs is turning out to be a novel regulatory mechanism, modulating diverse plant responses to various abiotic stresses. However, the information on such a crosstalk has not been schematically presented so far. Therefore, the present review aims to present the evidence that supports the crosstalk among ET, NO, and PAs during various abiotic stress conditions. The information presented in this review provides a mechanistic understanding of how these regulatory molecules affect the biosynthesis of different components and their associated signaling in tailoring the abiotic stress resilience in plants.

## ET, NO, and PAs: Interconnection and biosynthetic regulation

### Biosynthesis of ethylene

**Fig. 1:**
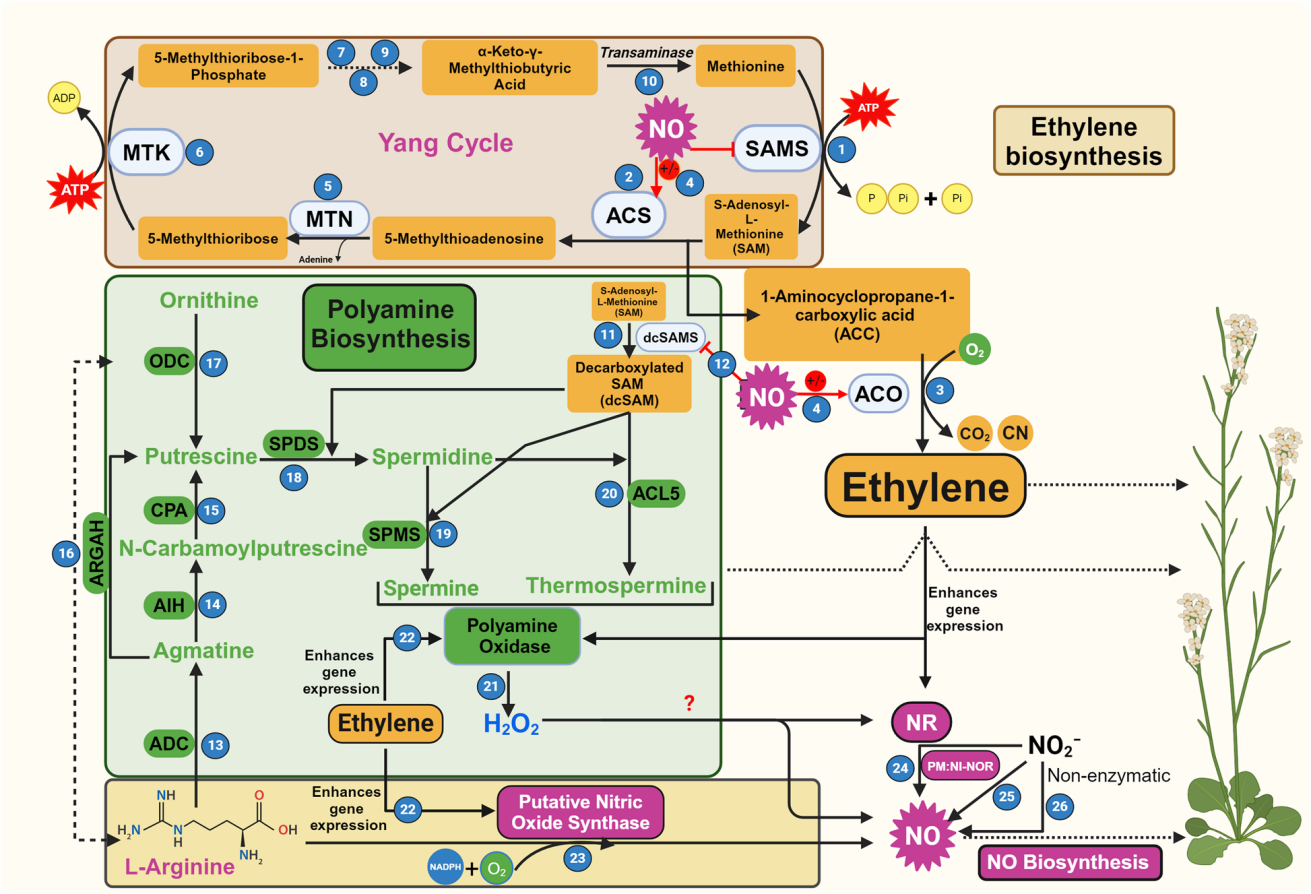
Biosynthetic pathways for ethylene (ET), nitric oxide (NO), and polyamines (PAs) and their interconnected regulation in plants. (1) The general precursor of ET, *S*-adenosyl-l-methionine (SAM) is formed by SAM synthetase (SAMS) using methionine at the expense of ATP. (2,3) SAM is utilized for ET and PA biosynthesis as per their demands. SAM gets converted into 1-Aminocyclopropane-1-carboxylic acid (ACC) through ACC synthase (ACS) and then to ET by ACC oxidase (ACO) by consumption of O_2_, forming CO_2_ and CN^−^ as by-products. (4) ACS and ACO activities are regulated by NO through S-nitrosylation. (5-10) The Yang cycle functions to recycle 5′-methylthioadenosine (MTA), a by-product of ACC biosynthesis, back to methionine. (5) MTA is converted to 5′-methylthioribose (MTR) by MTA nucleosidase (MTN) releasing adenine. (6) MTR is then phosphorylated by MTR kinase (MTK) into MTR-phosphate (MTR-P). (7-9) This intermediate is subsequently isomerized by MTR-P isomerase (MTI) to produce 5′-methylthioribulose-1-phosphate (MTRu-P) which is then converted to 1,2-dihidroxy-3-keto-5′-methylthiopentene (DHKMP) by the enzyme dehydratase-enolase-phosphatase (DEP) in a single step and finally to 2-keto-4-methylthiobutyrate (KMTB) by acireductone dioxygenase (ARD). (10) Methionine, the precursor for ET biosynthesis, is formed by the transamination of KMTB. (11) SAM also gets converted to decarboxylated SAM by the dcSAMS enzyme that provides the intermediate for PA synthesis. (12) NO also regulates dcSAMS activity. (13-15) During PA biosynthesis, arginine is converted to Putrescine (Put) in a series of reactions catalyzed by arginine decarboxylase (ADC), agmatine iminohydrolase (AIH), and N-carbamoyl putrescine amidohydrolase (CPA). (16) Arginine amidinohydrolyase (ARGAH) converts agmatine to Put, or hydrolyse arginine to ornithine (Orn). (17) Conversion of Orn to Put is catalyzed by Ornithine decarboxylase (ODC). (18–20) Put is also used for the biosynthesis of Spermidine (Spd) by Spd synthase (SPDS) which is further converted to the Spermine (Spm) and Thermospermine (tSpm) by Spm synthase (SPMS) and Thermospermine synthase (*ACL5*), respectively. (21) Polyamine oxidases (PAOs) oxidize Spd, Spm, and tSpm to produce different small molecular weight amines and a common by-product H_2_O_2_. (22) ET induces gene expression of PAOs to promote PA catabolism and (23) putative nitric oxide synthase (NOS) that leads to NO biosynthesis using L-Arginine as substrate in the presence of O_2_ and NADPH. (24–25) Nitrate reductase (NR) and Plasma membrane-bound Nitrite-Nitric oxide reductase (PM:Ni-NOR) use NO_2_⁻ as a substrate to generate NO. (26) Non-enzymatic sources are also reported to generate NO in vivo. ± : Positive or negative regulation

ET is a well-recognized phytohormone, known for regulating various plant processes including seed germination, root initiation, fruit ripening, leaf senescence, and stress responses along with its well-known “triple response” (Binder 2020). ET biosynthesis is relatively straightforward and involves only three enzymatic steps (Fig. 1) (Abeles et al. 2012; Riyazuddin et al. 2020). In the first step, sulfur-containing amino acid, methionine is converted into the precursor molecule S-Adenosyl methionine (SAM, an activated form of methionine), by the action of enzyme SAM synthetase (SAMS). SAM is then converted to 1-aminocyclopropane-1-carboxylic acid (ACC) by the action of ACC synthase (ACS) which is finally oxygenated to ET by the action of ACC oxidase (ACO) along with the release of CO_2_ and cyanide as by-products (Fig. 1) (Adams and Yang 1981; Pattyn et al. 2021). The 5-methylthioadenosine, generated as a by-product of ACS activity, is recycled back to methionine by the Yang cycle in a series of enzymatic steps (Fig. 1) (Pommerrenig et al. 2011). Overall, tight regulation of ET biosynthesis is exercised at both ACS and ACO enzymes at transcriptional and post-transcriptional levels (Zhao et al. 2021a). As ACS is a rate-limiting enzyme of ET biosynthesis, it is also termed as ACC-dependent pathway (Xu et al. 2021).

### Biosynthesis of nitric oxide

NO is another gasotransmitter involved in the regulation of various plant processes such as the breakdown of seed dormancy, stomatal movement, photosynthesis, senescence, floral regulation, seed germination, lateral and adventitious root formation, regulation of cellulose content in roots, and responses to stress conditions (Bethke et al. 2004; Wani et al. 2021; Zhang et al. 2023). NO in plants can be generated in various subcellular locations ranging from cytoplasm, chloroplasts, mitochondria, and peroxisomes by multiple enzymatic and non-enzymatic pathways through oxidative and reductive routes (Jahan et al. 2021). The oxidative pathways and their enzymes include mammalian nitric-oxide synthase (NOS)-like enzyme that converts L-arginine into L-citrulline and NO using NADPH and O_2_ (Fig. 1) (Corpas et al. 2009). Though NOS-like activity has been detected in multiple plants, no dedicated enzyme has been identified in higher plants so far (Allagulova et al. 2022; Corpas et al. 2022; Talwar et al. 2012). Another oxidative route for NO generation is the oxidation of hydroxylamines (Rümer et al. 2009). Recently, a novel oxidative route for NO generation has been uncovered in *Medicago truncatula* (López-Gómez et al. 2024)*.* This route involves the oxidation of oximes (indole-3-acetaldoxime (IAOx), a precursor of indole-3-acetic acid) by the activity of peroxidase (POD). This reaction is stimulated by the flavins such as riboflavin, FAD, and FMN and is inhibited by superoxide dismutase (SOD) (López-Gómez et al. 2024).

The reductive pathways for the NO generation include nitrate reductase (NR) and plasma membrane-bound nitrite reductase (PM-Ni:NOR) (Fig. 1) (Astier et al. 2018; Corpas et al. 2009; Durner et al. 1998; Talwar et al. 2012). NR-mediated NO generation is considered as the main reductive enzyme-based pathway in higher plants which generate NO as a by-product while performing its main function of converting nitrate (NO_3_¯) into nitrite (NO_2_¯) (Fig. 1) (Jeandroz et al. 2016). NR also helps in NO generation by supplying nitrite to other enzymes located on the plasma membrane (PM-Ni:NOR) (Stöhr et al. 2001). Additionally, xanthine oxidoreductase (XOR) and aldehyde oxidase (AO), collectively known as "non-dedicated nitrite reductases molybdoenzyme", also catalyze the nitrite reduction to NO (Maia and Moura 2018).

Along with these enzyme-mediated NO production pathways, non-enzymatic NO generation has also been reported in plants, especially at acidic pH (4.5) of the apoplast, where dismutation of NO_2_^−^ to NO and nitrate, and reduction of NO_2_^−^ to NO by ascorbic acid has been reported (Fig. 1) (Henry et al. 1997). Besides, non-enzymatic NO generation has also been reported from β-Carotene in the presence of light (Cooney et al. 1994; Tun et al. 2006).

### Biosynthesis of polyamines

**Fig. 2:**
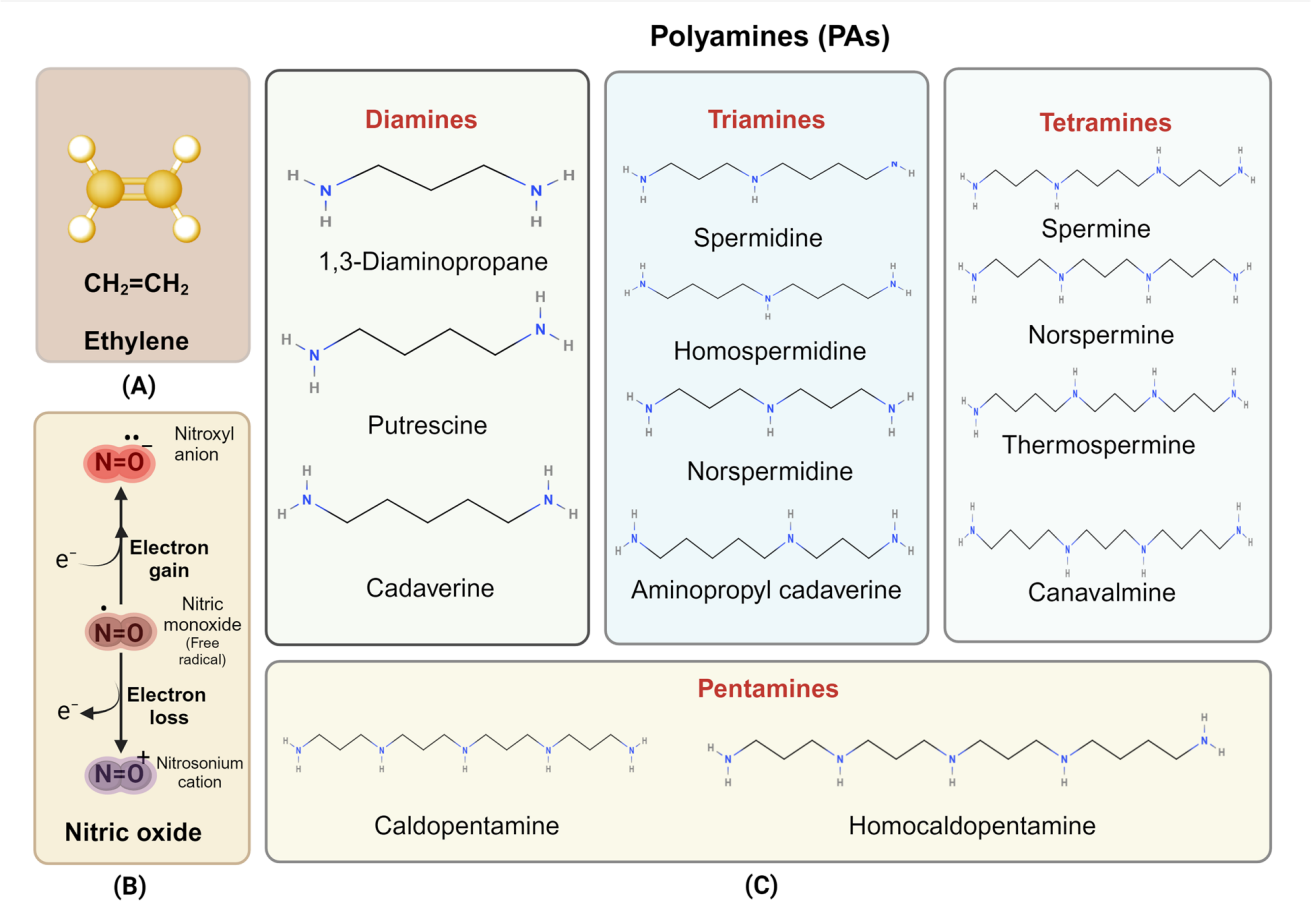
Molecular structures of (**A**) Ethylene, (**B**) Nitric oxide (NO), nitroxyl anion (NO^−^), and nitrosonium cation (NO^+^); and (C) different polyamines found in plants. Diamines: 1,3 diaminopropane, Putrescine, cadaverine; Triamines: Spermine, Homospermidine, Norspermidine, Aminopropyl cadaverine; Tetraamines: Spermine, Norspermine, Thermospermine, Canavalamine; Pentaamines: Caldopentamine, Homocaldopentamine

In contrast to ET and NO which are gaseous molecules, PAs are a group of small, aliphatic, basic, polycationic molecules comprised of variable hydrocarbon chains and amino groups. PAs are known to regulate diverse plant processes including cell division, root initiation, embryogenesis, flower development, fruit ripening, and stress tolerance (Asija et al. 2023; Jangra et al. 2023; Nandy et al. 2023; Tyagi et al. 2023). In plants, major PAs include putrescine (Put; diamine), spermidine (Spd; triamine), and spermine (Spm; tetraamine) (Fig. 2) (Chen et al. 2019; Jiménez-Bremont et al. 2022). In addition, several uncommon PAs, such as homospermidine, norspermidine, norspermine, thermospermine (tSpm), and cadaverine (Cad) have also been identified (Fig. 2) (Kuehn et al. 1990). Due to their polycationic nature, PAs can bind to anionic cellular constituents such as DNA, RNA, phospholipids, and acidic proteins to regulate their structure and stability in response to internal and external cues (Tiburcio et al. 2014). Since PAs are more abundant in plants than phytohormones, they control biological responses at millimolar concentrations (mM), implying that these may not have a truly hormonal role (Zahid et al. 2023).

Put is the principal PA which is synthesized by the decarboxylation of either L-arginine or L-ornithine, catalyzed by arginine decarboxylase (ADC) or ornithine decarboxylase (ODC), respectively (Fig. 1) (Walters 2003). Put is then converted to Spd by spermidine synthase (SPDS) which is further converted into spermine (Spm) by spermine synthase (SPMS) (Fig. 1) (Vuosku et al. 2018). Both of these conversions may utilize decarboxylated SAM (dcSAM) which is formed from SAM by the action of the enzyme S-adenosylmethionine decarboxylase (SAMDC) (Fig. 1) (Napieraj et al. 2020). As ODC in plants is usually less active than ADC, arginine decarboxylation is seen as the major pathway for Put biosynthesis (Shao et al. 2022). PAs are catabolized by polyamine oxidases (PAOs) to produce different small molecular weight amines and a common by-product H_2_O_2_ (Benkő et al. 2022).

### ET-NO-PAs: biosynthetic crosstalk

The biosynthesis of ET, NO, and PA is interestingly interconnected through the specific and/or common precursors and regulatory mechanisms in such a way that the biosynthesis of one can affect the synthesis of the other (Fig. 1). ET synthesis is very selective and is solely dependent on SAM, which also serves as a precursor for PA (Fig. 1). Since SAM is the common precursor for both ET and PA, under the conditions of limited SAM availability, ET and PA biosynthetic pathways may compete for this shared common substrate (Nambeesan et al. 2008). For instance, inhibition of ACC synthesis, a direct precursor of ET, results in increased PA (Rauf et al. 2021). Conversely, inhibition of PA biosynthesis increases concentrations of ACC and ET, implying that one SAM‐dependent pathway is stimulated when the other is blocked (Rauf et al. 2021). Using ^14^C-radiolabelled methionine, the diversion of SAM for the ET and PA synthesis was investigated in tomato fruits (Lasanajak et al. 2014). The results suggested that the PA synthesis from SAM declines in the later stages of ripening when the ET level increases (Lasanajak et al. 2014). However, transgenic expression of a yeast *ySAMDC* gene reversed this decline in PAs without affecting the ET concentrations. These results suggest that the cellular flux of SAM is allocated based on its demand for competing pathways for ET or PAs synthesis (Lasanajak et al. 2014). As PAs can also be synthesized by other precursors such as arginine or ornithine, PA biosynthesis could be switched to other alternate routes in the case of limited SAM availability (Chen et al. 2019). Of these, arginine also functions as a precursor for NO and regulates the enzyme SAMDC, an enzyme involved in PA synthesis (Lokesh et al. 2019; Napieraj et al. 2020). Thus, PA synthesis, on one side, overlaps with ET biosynthesis through common precursor SAM and on the other hand, overlaps with NO through arginine (Fig. 1).

In addition to sharing these common precursors, reports have also shown that the biosynthesis and signaling of one affects the synthesis of another that has no common precursor. For instance, the antagonistic relationship between NO and ET has been well documented and was first highlighted almost eight decades back when it was noticed that NO inhibits the formation of ET from ethane (Burnham and Pease 1940). Following this, multiple reports further validated their antagonistic relationship during fruit ripening, leaf and floral senescence, dark-induced stomatal closure, and cadmium-induced cell death (Kolbert et al. 2019; Manjunatha et al. 2012; Mur et al. 2009; Niu and Guo 2012; Song et al. 2011). Nonetheless, synergistic interactions between these two have also been reported during UV-B-induced stomatal closure and iron deficiency-induced expression of iron acquisition genes (García et al. 2011). Notably, ET is known to induce NO biosynthesis by triggering the expression of NOS and NR (Hou et al. 2013; Xu et al. 2017). These results suggest that the relationship between these two gasotransmitters may depend upon a variety of factors including environmental conditions, upstream signals, and genetic regulation. Exogenous NO negatively alters the expression and activities of ACS and ACO which affects ACC accumulation, culminating in low ET production (Fig. 1) (Lokesh et al. 2019; Singh and Bhatla 2018). Such ET inhibition probably works by NO-mediated S-nitrosylation of ACS and ACO or by the formation of a binary ACO-NO complex. This binary complex subsequently chelates the ACC, forming a stable ternary complex, the ACC–ACO–NO complex, leading to declined ET generation (Manjunatha et al. 2012; Zhu et al. 2006). This crucial information has been exploited commercially to enhance the shelf-life of fruits by applying NO donors which extends their shelf life from 14 to 120 days (Manjunatha et al. 2010).

Along with inhibiting ET biosynthesis, exogenous NO treatment also induces the biosynthesis of PAs as observed in banana plants (Lokesh et al. 2019). However, SAMDC, the enzyme that participates in PA biosynthesis through SAM, was downregulated, suggesting that ET and PA do not compete for SAM (Lokesh et al. 2019). Subsequent experiments suggested the existence of an alternate route for PA synthesis through ADC (Lokesh et al. 2019). These experimental findings lead to two speculations: (1) high NO concentrations, likely during stress conditions, induce PA synthesis from arginine through ADC and/or ODC; and (2) under normal conditions when endogenous NO level is low, diversion of SAM occurs for ET or PA synthesis as per the cellular requirement. SAM, the common substrate for ET and PAs, is produced by the activity of the SAMS enzyme (Fig. 1). In Arabidopsis, three isoforms of SAMS i.e., SAMS1, SAMS2, and SAMS3 are available of which only SAMS1 is regulated by S-nitrosylation at Cys-114. S-nitrosylation of SAMS1 inhibits its activity while the absence of Cys-114 residue in the other two isoforms makes them insensitive for inhibition (Lindermayr et al. 2006) (Fig. 1). This differential response suggests that only SAMS1 is amenable to NO-mediated inhibition of SAM biosynthesis. Therefore, it can be construed that at least partial control of SAM-mediated synthesis of ET and PA is exercised by NO. Thus, the S-nitrosylation process acts as a key regulatory control on ET biosynthesis in plants by targeting both SAMS1 (Lindermayr and Durner 2009; Lindermayr et al. 2006) and ACO (Liu et al. 2023).

## ET, NO, and PAs: signaling, crosstalk, and abiotic stress tolerance

### UV-B stress

**Fig. 3:**
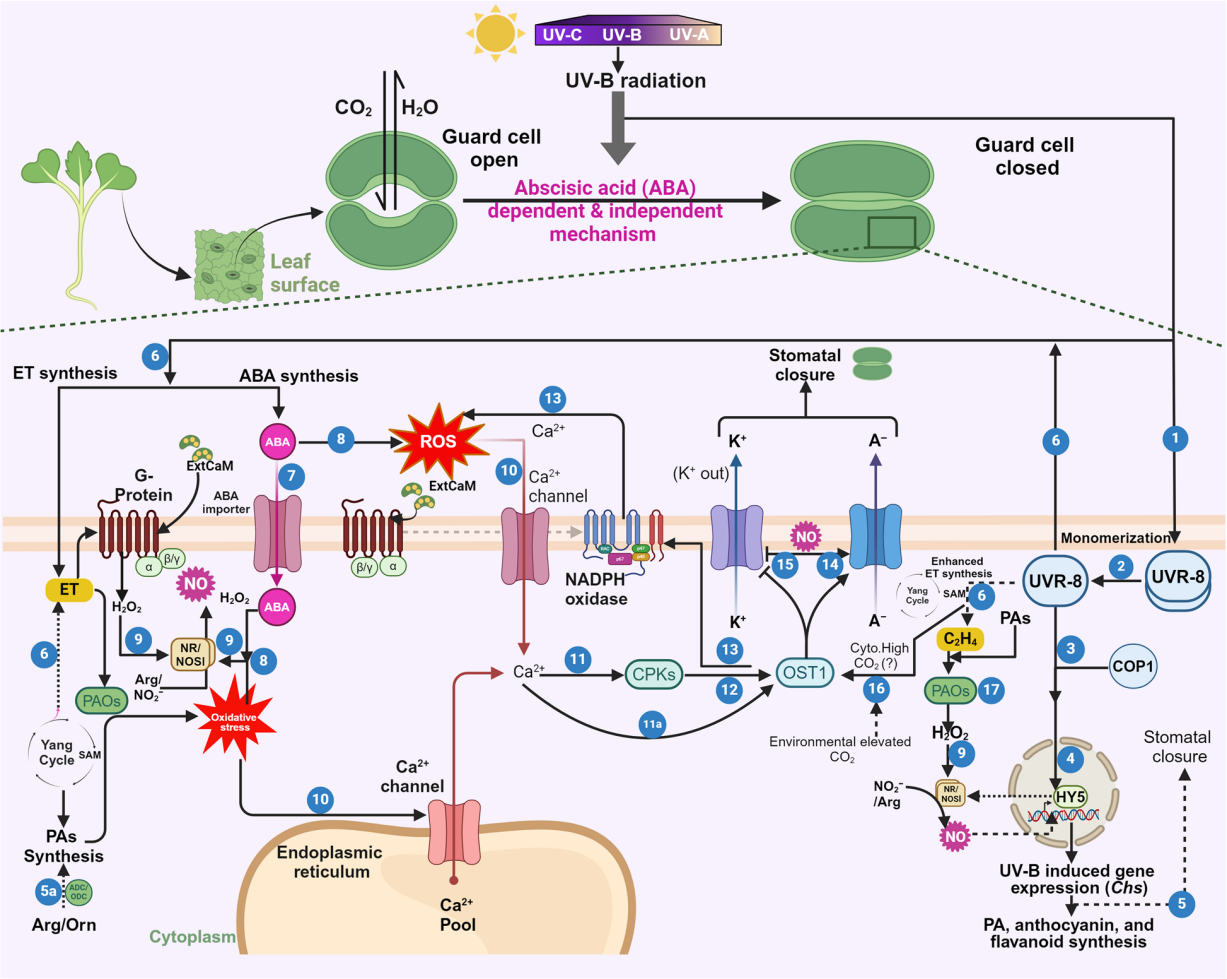
Schematic representation illustrating the interplay between the UV-B radiation and responses among ethylene (ET), nitric oxide (NO), and biologically active polyamines (PAs). (1–2) Plants perceive UV-B radiation through a specialized photoreceptor, Ultraviolet resistance locus 8 (UVR8), which undergoes monomerization. (3) The UVR8 monomer interacts with CONSTITUTIVELY PHOTOMORPHOGENIC1 (COP1), a ubiquitin ligase (3), and hinders its ability to poly-ubiquitinate the transcription factor ELONGATED HYPOCOTYL5 (HY5). (4) As a result, HY5 is spared from degradation, and its levels increase. (5) Together, UVR8 and COP1 orchestrate UV-B-induced photomorphogenesis and stress acclimation in plants by stomatal closure, UV-B induced gene expression (for flavonoid, anthocyanin, and polyamine synthesis. (6) When exposed to UV-B, UVR8 triggers the generation of molecules like ABA, NO and ET. (7) ABA is internalized in guard cell cytoplasm through an ABA importer. (8–9) ABA causes reactive oxygen species (ROS) generation and ROS/H_2_O_2_-induced NO synthesis through NR/NOS-like enzymes. (11, 11a-12) Increased ROS concentrations result in the opening of Ca^2+^ channel (10) and cytosolic Ca^2+^ dependent accumulation, leading to activation of CPK and independent pathway regulating Open Stomata 1 (OST1) cascade downstream. (13) Activated OST1 also induces membrane-bound NADPH oxidase to further contribute to the ROS pool. (14–15) Activated OST1 and cytosolically synthesized NO cause activation of anion channels and inhibition of cation (K^+^) channels leading to stomatal closure which limits water loss and prevents cell damage. (16) The UV-B radiation also causes ET synthesis through the UVR8 signaling module. (17, 9) Synthesized ET follows the ET signaling pathway and triggers the synthesis of H₂O₂ through polyamine oxidase (PAOs) which then induces the generation of another signaling molecule, NO via activation of nitrate reductase/ nitric oxide synthase-like (NR/NOSl) enzyme. *Chs*: Chalcone synthase

UV-B is a high-energy, non-ionizing radiation that is perceived by the plants as a part of the electromagnetic spectrum from the sun (Yemets et al. 2015). High and continuous doses of UV-B exposure induce changes in various morphological and physio-biochemical parameters and are known to be lethal for plants (Vanhaelewyn et al. 2020; Wang et al. 2006). UV-B is perceived by plants through a specialized photoreceptor, Ultraviolet resistance locus 8 (UVR8), that undergoes monomerization upon UV-B absorption (Podolec and Ulm 2018). UV-B radiation tolerance in plants occurs through direct regulation of flavonoid biosynthesis through various transcription factors (TFs) such as ELONGATED HYPOCOTYL 5 (HY5), CONSTITUTIVELY PHOTOMORPHOGENIC1 (COP1), BRI1 EMS SUPPRESSOR 1 (BES1), and MYB-domain proteins involving mitogen-activated protein kinases (MAPKs) signaling (Shi and Liu 2021). The interaction between UVR8 and COP1 leads to stomatal closure as well as changes in gene expression of important genes such as chalcone synthase resulting in stress acclimation in plants (Podolec and Ulm 2018). This UV-B stress-induced stomatal closure in plants takes place majorly because of the activation of ABA-dependent signaling which causes H_2_O_2_ generation (Fig. 3) (Tossi et al. 2009). Increased ROS leads to the opening of the Ca^2+^ channel and subsequent increase in the cytosolic Ca^2+^ which triggers Open Stomata 1 (OST1). Activated OST1 induces the activation of anion channels and inhibition of cation (K^+^) channels to induce stomatal closure (Fig. 3).

Intriguingly, emerging evidence suggests the potential regulatory roles of ET and NO along with ABA in UV-B-induced stomata closure (Fig. 3) (Kolbert et al. 2019). ET triggers H_2_O_2_ synthesis by inducing the POD activity instead of NADPH oxidase during UV-B stress (He et al. 2011). Exogenous ET application also induces H_2_O_2_ production in guard cells resulting in subsequent stomatal closure, which further confirms the role of ET in this process (He et al. 2011). Another line of evidence suggests that UV-B radiation also induces NO, ABA, and ROS along with ET. This UV-B-induced NO is synthesized through NOS-like enzyme activity in the guard cells which is preceded by stomatal closure (He et al. 2005). However, the fact that the use of ET inhibitors did not affect ROS accumulation and NOS activity, and scavenging ROS or exogenous NO application promote the synergistic ET accumulation suggests that both ROS and NO function upstream of ET and play an important role in UV-B-induced ET synthesis (Wang et al. 2006). However, some other studies show that ET participates in inducing stomata closure during UV-B stress by activating the Gα subunit, GPA1, via ROS and NO production (Fig. 3) (Li et al. 2022). Moreover, UV-B stress-induced ET synthesis also triggers the accumulation of PAs and ABA. In contrast, ABA treatment decreases the UV-B-induced synthesis of ET and PAs, especially Spm and Spd, but interestingly enhances the Put content (Rakitin et al. 2009). These results suggest that ABA probably reduces the cellular SAM content, resulting in the reduction of both ET and Spm/Spd. Nonetheless, the increased content of the Put in response to ABA treatment under UV-B stress could be due to the activation of an alternate pathway for PA synthesis originating from ornithine or arginine instead of SAM (Walters 2003). These results also suggest that ET functions upstream of both ABA and PAs to induce closure of stomata and other UV-B-induced responses (Rakitin et al. 2009). These speculations were further validated by subsequent studies which showed Put accumulation in plants along with the loss in Spd and Spm pools in response to exogenous ET treatment. However, these ET-mediated alterations in PAs were less pronounced in the *etr1-1* mutants which exhibit impaired ET perception (Prudnikova et al. 2019). Treatment with 1-MCP (ET production inhibitor) before and 24 h after the irradiation, enhanced plant sensitivity to UV-B and Put accumulation which further confirmed the key role of ET and downstream PAs in UV-B stress tolerance (Prudnikova et al. 2019). Therefore, it seems that multiple routes for stomatal regulation work in plants in response to UV-B stress (Soheila et al. 2001). These findings highlight a complex interplay among UV-B radiation, ABA, ET, PA, and NO for stomatal regulation. Notably, H_2_O_2_ seems to be the connecting and common link among these molecules which might be responsible for downstream action (Fig. 3).

### Low-temperature stress

**Fig. 4:**
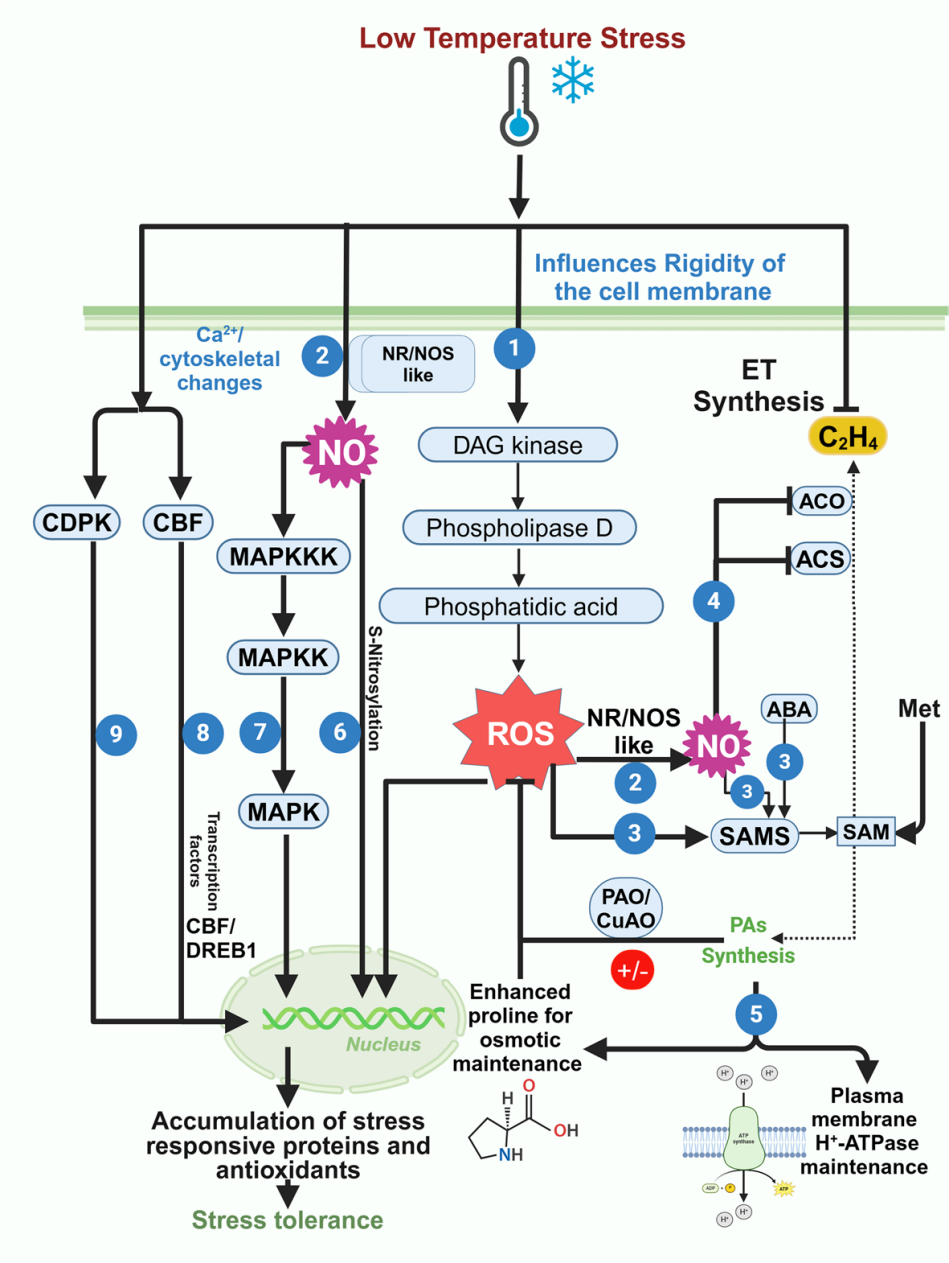
Schematic representation showing crosstalk among ethylene (ET), nitric oxide (NO), and polyamines (PAs) in plants for cold stress tolerance. (1) Cold stress damages the plasma membrane and triggers lipid-based signaling involving DAG kinase, Phospholipase D (PLD), and Phosphatidic acid (PA), leading to an oxidative burst. (2–3) The H_2_O_2_ induces NR/NOS-based NO biosynthesis, which together with Abscisic acid (ABA) and ROS act positively on SAM synthetase (SAMS) to promote PA biosynthesis. (4) Enhanced NO also causes suppression of ET synthesis by inhibiting ACS and ACO enzymes. (5) PAs help in correcting plant cellular machinery by augmenting membrane stability, preventing ion leakage, and scavenging ROS. (6–7) Cold stress activates the expression of cold-responsive genes either by S-nitrosylation of the target proteins or by mediating the response through the MAPK cascade. (8–9) Cold stress tolerance is also executed in plants by changes in Ca^2+^ /cytoskeleton either mediating signal through cold binding factors (CBF) or CDPKs

Low-temperature stress, broadly subcategorized as chilling (0–15 ºC) and freezing stress (< 0 °C), primarily impacts plasma membranes and transforms them into rigid structures that impair their functions (Fig. 4) (Aslam et al. 2022; Ding and Yang 2022). Plants have evolved various strategies to sense temperature changes and augment stress tolerance (Gupta and Deswal 2014). Cold stress tolerance is acquired either by the activation of genes that are responsive to C-repeat binding factors/dehydration-responsive element binding protein 1 s (CBF/DREB1s) through several TFs or by the direct action of NO through S-nitrosylation which regulates several downstream processes including ET synthesis (Fig. 4) (Sougrakpam et al. 2023). In addition, PAs, especially SAM-derived PAs, are also known to augment cold tolerance by binding to the membrane phospholipids to prevent cytolysis and by inducing the apoplastic H_2_O_2_ production which is positively correlated with their oxidation (Fig. 4) (Guo et al. 2014; Li and He 2012). Studies involving exogenous application of PAs have shown that PA treatment promotes NO generation by inducing the activities of NR and NOS-like enzyme through an H_2_O_2_-dependent pathway under cold stress (Diao et al. 2017). Notably, heterologous expression of *SAMS1* from *Medicago sativa* in tobacco resulted in elevated SAM, however, no significant changes in the levels of PAs and ET were observed. The transgenic plants showed higher transcript levels for both synthesis and oxidation of PAs which suggests that the SAM produced because of the *SAMS1* overexpression is utilized for the biosynthesis of PAs that subsequently get oxidized, thereby making no change in cellular PAs level as compared to wild-type (Guo et al. 2014). Therefore, it can be deciphered that cold tolerance is predominantly governed by PA catabolism, leading to the generation of H_2_O_2_ that further induces other signaling molecules such as NO (Fig. 4) (Pattyn et al. 2021; Zhu et al. 2006).

Along with PAs, an elevated synthesis of ET has also been shown in several plants including cucumber (Wang 1987) and Zoysia grass (*Zoysia japonica*) (Sun et al. 2020). However, transgenic lemon plants expressing a micro RNA, miR396b, from *Poncirus trifoliata* showed enhanced cold tolerance owing to the suppression of ET synthesis and induction of PA synthesis (Zhang et al. 2016). Exogenous application of PA also delays the stimulation of ET with decreasing temperature while maintaining the plasma membrane H^+^-ATPase and enhancing the proline content that imparts stress tolerance (Jankovska-Bortkevič et al. 2020). Although the exact role of ET in cold stress tolerance is not well understood, it is possibly associated with the activation of downstream TFs that bind to the CBF promoter to regulate cold stress tolerance. Overall, these evidences, although limited, highlight an intricate regulatory but interconnected relationship among PAs, ET, and NO under cold stress tolerance. However, the interaction of PAs either with ET or NO has been poorly investigated under cold stress and requires further exploration.

### Salt stress

**Fig. 5:**
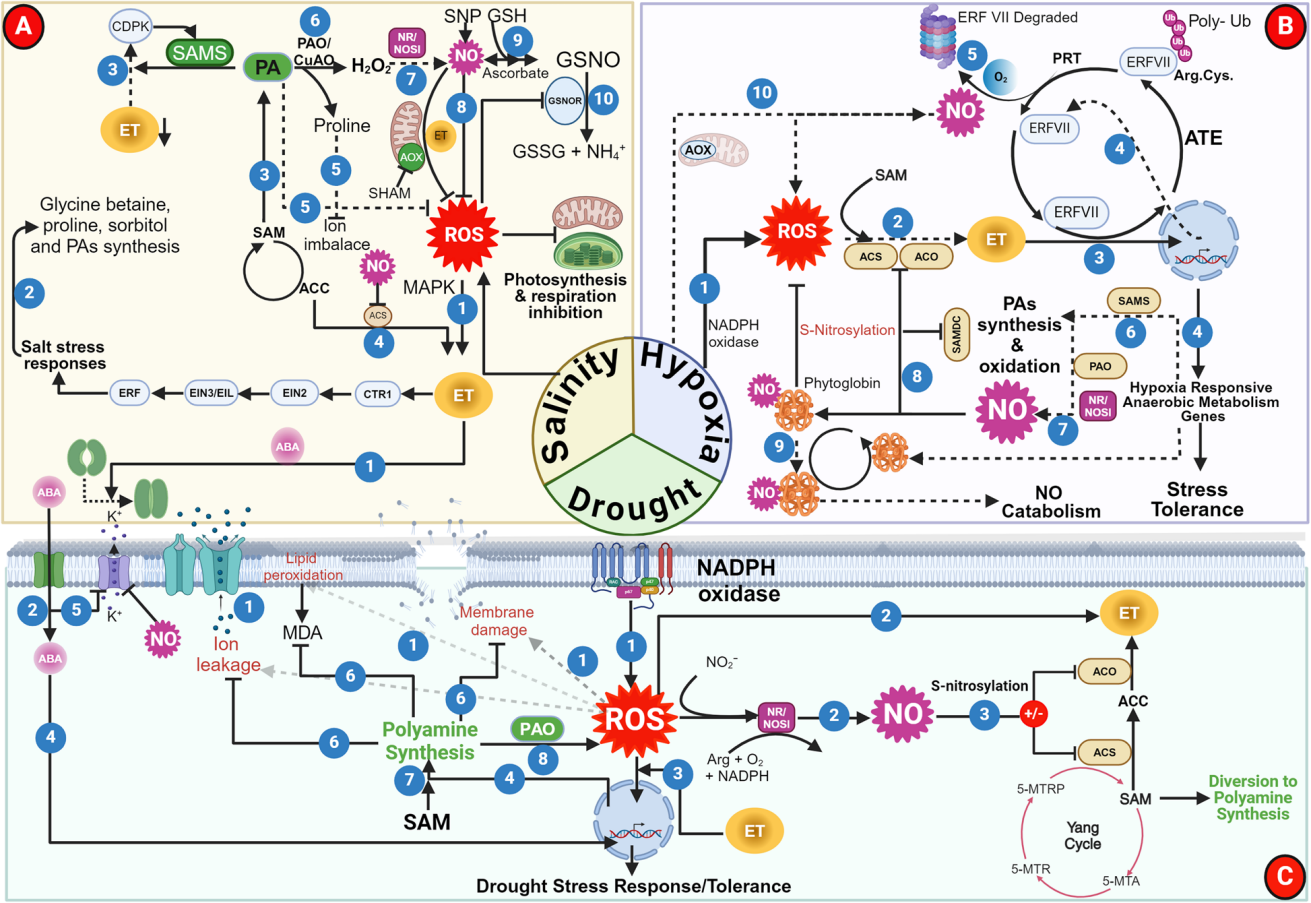
Schematic representation illustrating the interplay of ethylene (ET), nitric oxide (NO), and polyamines (PAs) in salinity, hypoxia, and drought stresses in plants. **A** (1) Salinity causes cellular oxidative bursts and initiates the ET-ABA interaction and related signaling to induce stomatal closure. (2) Expression of salinity stress-induced genes results in the synthesis of osmolytes such as glycine betaine, sorbitol, and proline that maintain cellular ion homeostasis. (3) On the other hand, low ET level upregulates PA synthesis via CDPK-mediated signaling through SAMS, or unutilized SAM is diverted to PA synthesis. (4) A low level of ET is possibly ensured by the regulation of ET synthesizing enzyme ACS by NO through S-nitrosylation. (5) The PAs help directly in salinity stress mitigation by ROS scavenging or indirectly by promoting proline synthesis and maintaining cellular ion homeostasis. (6–7) To maintain cellular PA level, excess PAs are oxidized by polyamine oxidases (PAOs), generating H_2_O_2_ that induces NO synthesis through activation of NR/NOS enzymes. (8) NO also helps in re-functionalizing the organelles like mitochondria and chloroplast that exhibit reduced activities due to oxidative stress. (9) NO is also stored for rapid response as S-Nitrosoglutathione (GSNO) (sequestered with reduced glutathione, GSH) and released instantly as and when required. (10) Excess GSNO is removed by GSNO reductase (GSNOR) whose level is finely regulated by in vitro ROS pool. Salicylhdroxamic acid (SHAM), an inhibitor of alternate oxidase (AOX), acts by blocking the uninhibited flow of electrons through AOX. SNP: NO donor. **B** (1) Hypoxia tolerance involves ET and NO as key regulating molecules. (2) Hypoxia induces oxidative burst through NADPH oxidase that activates ET synthesis. (3) It leads to enhanced gene expression related to hypoxia/anaerobic metabolism through ET-augmented stability of a hypoxia-specific group of transcription factors called ERFVII. (4) The ET-mediated NO depletion leads to the accumulation of ERFVII as ET provides stability to these proteins. Stabilized ERFVII moves to the nucleus where it helps in the upregulation of genes such as SAMS which participates in PA synthesis, and the hypoxia/anaerobic metabolism-related genes. (5) The presence of NO or O_2_ makes ERFVII unstable and induces its degradation by the N-degron pathway. (6–7) NO synthesis is also upregulated via activation of NR/NOS-like protein following PA synthesis, probably through H_2_O_2_ generated by PA oxidation mediated by PAOs. (8) NO acts as a key regulating molecule inhibiting enzymes involved in ET (ACO, ACS) and PA synthesis (SAMS) possibly through S-nitrosylation. (9) NO also augments hypoxia tolerance by enhancing the activity of PHYTOGLOBIN1 (PGB1), which scavenges cellular NO that contributes to ERFVII destabilization. (10) Hypoxia is also regulated by induction of alternative oxidase (AOX), a terminal oxidase enzyme, present in mitochondria, associated with hypoxia tolerance by helping in NO generation. **C** (1) Drought stress induces NADPH oxidase-dependent oxidative burst, leading to membrane damage, lipid peroxidation, ion leakage, and osmotic changes. (2–3) In response to this, signaling molecules such as ABA and NO are synthesized but ET is found to be downregulated through S-nitrosylation of key ET synthesizing enzymes (ACS, ACO). (4–5) Synthesized ABA is imported into the cell via an ABA importer that causes regulation of various drought stress-related gene expressions (including PAs) and inhibition of the K^+^ channel that causes stomatal closure to prevent further loss of water. (6) Upregulated PAs synthesized in response to drought stress help in quenching ROS, preventing ion leakage, ameliorating membrane damages as well as lipid oxidation. (7,3) Increased PA synthesis during drought stress is mainly derived from the SAM pool as ET remains downregulated during drought stress. (8,2) Elevated level of PA is oxidized by polyamine oxidases (PAOs) generating H_2_O_2_ that further induces synthesis of NO through NR/NOS-like enzyme ensuring well-coordinated regulation among ET-NO-PAs. Abbreviations: ATE, Arg-tRNA transferase; PRT, Proteolysis; poly-Ub, poly-ubiquitin chain

Increasing soil salinity because of poor irrigation practices imposes salt stress on the plants, leading to ion imbalance, osmotic homeostasis, and oxidative stress in plants that alter key physiological processes including photosynthesis and respiration (Zhao et al. 2021b). PAs are at the forefront during salinity stress as they mitigate stress by preventing membrane damage and maintaining ion homeostasis due to their polycationic nature (Chen et al. 2007; Roy et al. 2005). As a general trend, salinity decreases Put levels while increasing Spm and/or Spd that confer salinity tolerance (Fig. 5A). Decreased Put levels suggest its catabolism through diamine oxidase (DAO) which can contribute to proline accumulation that further helps in the maintenance of cellular ion homeostasis (Bouchereau et al. 1999). This increased PA synthesis during salinity stress is likely from its precursor SAM as enhanced *SAMS* expression has been reported during salinity stress in multiple plants. The production of PAs from SAM also ensures reduced ET biosynthesis which is crucial for salinity stress tolerance. The activation of SAMS (*SAMS1*) is potentially associated with a calcium-dependent protein kinase (CDPK) family protein CDPK6. Overexpression of the cucumber *CDPK6* gene in tobacco resulted in decreased H_2_O_2_ and ET levels under salt stress along with the reduction in stomata density and lipid peroxidation compared to wildtype which significantly improved salt tolerance. In contrast, silencing of *CDPK6* promoted ET synthesis which reduced salt tolerance. These results imply that *CDPK6* overexpression inhibits ET biosynthesis, leading to the diversion of SAM for PA synthesis which reduces lipid peroxidation and further oxidizes to produce H_2_O_2_. Although the PA content was not measured in this study, the obtained results certainly suggest that salt tolerance is achieved by diverting SAM for PA synthesis rather than ET to ensure osmotic homeostasis. This reiterates that the fine-tuning of ET-PA biosynthesis regulates salinity stress tolerance in plants (Zhu et al. 2021).

The cross-talk between ET and NO has also been observed during salinity stress. It is hypothesized that ET biosynthesis takes place at the initial stages of salt stress which goes down at the later stages where accumulation of PAs occurs. During the early stages, ET upregulates TF EIN2 via activation of CTR1, EIN3/EIL, and ERF to activate downstream signaling that promotes salt tolerance. However, ET accumulation for a longer duration downregulates the expression of gene ETR1/ETR2/EIN4 which delays salt tolerance (Fahad et al. 2015). ET-based salinity tolerance in plants also involves MAPK6 which probably mediates the crosstalk between ET and NO (Li et al. 2014). In the root apices and suspension culture cells of tomato plants, accumulation of both ET and NO was observed under salinity stress (Poór et al. 2014). However, Arabidopsis showed accumulation of ET alone which activates EIN2 TF. EIN2, a membrane protein and positive regulator of ET signaling is also known to shape salt stress responses in plants as a loss-of-function mutation in *EIN2* results in salt hypersensitivity (Lei et al. 2011). EIN2 shows interaction with another protein MA3 domain-containing protein (ECIP1) during salt stress which is also a regulator of ET signaling. ET also upregulates the synthesis of glycine betaine which helps in ion homeostasis to mitigate the toxic effects of salinity (Arif et al. 2020). To sum up, the action landscape of ET-NO-PAs in salinity stress tolerance in plants operates through two routes: first, ET and/or NO-mediated suppression of oxidative stress, and second, PAs or ET synthesis followed by PA oxidation, leading to H_2_O_2_ production that further causes NR mediated NO generation involving MAPK6, leading to suppression of the oxidative stress (Gechev and Hille 2005; Wang et al. 2010b). In addition to the NR-based NO generation, salinity stress-exposed plants also exhibit NO synthesis through NOS-like enzyme and inhibition of the GSNOR activity (Shang et al. 2022).

Alternate oxidase (AOX) enzymes are known for their key roles in promoting salt stress tolerance. These enzymes are non-energy conserving, terminal oxidases, that participate in the mitochondrial electron transport chain (Saha et al. 2016). The AOX enzyme regulates the synthesis and coordinates with important signaling molecules such as H_2_O_2_, O_2_^-^, and NO especially during salt and hypoxia stress conditions. Among these, NO acts as an upstream signaling molecule that triggers the *AOX* expression which subsequently lowers the oxidative stress and photosynthetic damage caused by salt stress. Accordingly, the application of the AOX inhibitor results in reduced salt stress tolerance (Jian et al. 2016). Along with the enhanced AOX expression, induction of the alternative respiratory pathway and enhanced ET emission have also been reported during salinity stress. The ET so produced is associated with the reduction of H_2_O_2_ and accumulation of pyruvate. In contrast, suppression of the alternative respiratory pathway results in severe cellular damage under salt stress (Wang et al. 2010a). These results suggest that NO and ET both participate in the regulation of AOX under salt stress to mitigate ROS-induced damage (Fig. 5A). Besides, NO is also known to regulate ET biosynthesis by affecting the activities of ET biosynthetic enzymes. In sunflower, the depletion of NO and salt stress together showed additive effects on the enhancement of ACO activity, resulting in reduced lateral root proliferation (Singh and Bhatla 2018). Another line of evidence suggests the involvement of ET signaling protein EIN3 in NO-induced salt stress mitigation in Arabidopsis seedlings. NO produced during salt stress stabilizes EIN3 protein which is a crucial component of ET signaling (Li et al. 2015). EIN3 is ubiquitinylated and is degraded by ubiquitin-mediated proteolysis under normal conditions, leading to the inhibition of ET signaling (Riyazuddin et al. 2020). Therefore, NO-mediated stabilization of EIN3 suggests activation of ET signaling which was further supported by the increased expression of *ACO4* and *ACS2* genes. However, NO does not seem to regulate the *ACO4* and *ACS2* expressions as their expression was solely induced by salt stress and not by NO donor treatment (Li et al. 2015). These results provide a further connection between NO and ET signaling mediated by EIN3 during salt stress. Although the exact mechanism behind NO-induced EIN3 stabilization is not known, the possible role of S-nitrosylation in this phenomenon can not be neglected. Overall, the relationship between ET and NO is difficult to define under salt stress and it is currently elusive which one acts upstream of the other. It is also reported that salinity stress leads to direct induction of ET or PA or both and these two in turn induce NO generation (Fig. 5A). Nonetheless, further experimentations are needed to validate these observations across different plants.

### Flooding/hypoxia stress

Flooding is detrimental to almost all higher non-aquatic plants. Flooding induces hypoxia due to limited underwater diffusion of gases, directly impacting aerobic respiration and consequent reduction of ATP production (Loreti and Perata 2020). Such conditions are primarily tailored by the ET-based signaling and defense system (Khan et al. 2020). ET accumulation is reported in multiple plants under flooding conditions which induces morphological and anatomical modifications to prevent hypoxia (Hartman et al. 2019; Voesenek and Sasidharan 2013). ERFVII, a plant-specific TF, acts as a critical regulator of plant survival under flooding and hypoxia conditions. Flooding-induced ET regulates the ERFVII activity and reduces NO in the cells. ERFVII is destabilized in the presence of cellular NO and O_2_, and the absence of either permits its accumulation and hypoxia-related responses (Fig. 5B). The stable ERFVII protein migrates to the nucleus where it activates the transcription of Hypoxia-Responsive Genes (HRGs) including those encoding for proteins required for alcoholic fermentation. ERFVII also enhances the expression of a NO scavenger protein, phytoglobin1 (PGB-1), that helps in regulating cellular NO levels. On the other hand, ET limits ERFVII proteolysis under normoxic conditions by enhancing the NO scavenger PGB-1 activity that lowers the cellular NO level. ERFVII group of TFs also interacts with the genes required for anaerobic metabolism (Fig. 5B) which in turn stabilize ERFVII and activate anaerobic responses at the transcriptional level (Gasch et al. 2016). The onset of anaerobic conditions triggers ROS burst via activation of NADPH oxidase and NO that subsequently shapes the ET biosynthesis through S-nitrosylation of ET synthesizing enzymes which augment hypoxia tolerance (Fig. 5B) (Park et al. 2022; Yamauchi et al. 2017). Transcriptomic analysis of flood-sensitive and flood-tolerant sweet potato cultivars revealed the upregulation of genes involved in ET, ROS, and NO synthesis under flooding stress, further confirming the accumulation of all these compounds. These molecules play a vital role against flooding stress and are likely to be interconnected through ERFVII (Fig. 5B).

Under anoxic conditions, ROS generation activates downstream signaling that regulates the expression of various genes belonging to the heat shock proteins (HSPs) and ROS-homeostasis, a mechanism that allows plants to survive longer during the absence of O_2_ (Pucciariello et al. 2012). This mechanism is likely not regulated via ET signaling component ERFs, implying the existence of an alternate route that facilitates plant survival under anoxia without involving ET signaling. However, the potential role of NO or PAs in this route cannot be neglected (Gibbs et al. 2014). For instance, the exogenous application of PAs (Spd) in flooding-sensitive bamboo (*Phyllostachys pracecox*) reduces oxidative damage induced from hypoxia stress by limiting ET and ROS generation, enhancing ABA and IAA levels, and activating the antioxidant enzymes, thereby alleviating the plant growth inhibition. The decreased ET emission in this case is linked with the downregulation of enzymes involved in ET biosynthesis such as ACO, ACS, and SAMDC (Gao et al. 2022). Hypoxia and anoxia are also known to affect the AOX at transcript, protein, or activity levels in the leaves or roots of several species (Vanlerberghe 2013). A study on tobacco plants overexpressing *AOX* showed increased transcripts of ACS, ACO, and ERF, indicating suppression of ET biosynthesis in comparison with the knockdowns under hypoxia. However, the concentration of ET was found to be increased along with the NO which suggests that the increased concentration of ET was because of NO-induced S-nitrosylation of ET biosynthesis enzymes which improved their activities, leading to ET accumulation. Indeed NO-induced S-nitrosylation was detected in ERFVIIs, cytochrome c-oxidase, aconitase, and ascorbate peroxidase that enhanced their activities (Zafari et al. 2022). In sweet potato, ET and PA levels were increased 4 days post-flooding treatment along with an accumulation of H_2_O_2_. ET production occurred in small amounts possibly by ACC transport from roots to leaves while PAs were accumulated because of the increased expression of *SAMS2*. The accumulated H_2_O_2_ due to hypoxia might further act as a signal for the induction of NO synthesis through NR activation as seen during flooding stress (Park et al. 2022). Nevertheless, more studies are certainly required to trace the relationship among ET-NO-PA and their their interactions with ROS in shaping flooding/hypoxia stress tolerance in plants (Fig. 5B).

### Drought stress

Drought is currently one of the most prominent threats influencing crop yield across the globe, especially in the tropical and subtropical regions (Riyazuddin et al. 2022). Drought stress causes a lowering of water content resulting in a change in osmotic status, altered ion concentrations, and oxidative stress (Khan et al. 2021). Plants retaliate to the drought conditions by synthesizing phytohormones such as ABA which helps in stomatal closure to avoid excess water loss. In the case of NO and ET, drought stress is known to inhibit their synthesis while PA, especially Spm, levels are increased (Cvikrová et al. 2012; Magalhaes et al. 2000). These trends in NO, ET, and PA levels have been reported in multiple plants including wildtype and transgenics. For instance, elevated PAs (mainly Spm and Spd) and reduced NO and ET levels were reported during enhanced drought stress tolerance in transgenic barley plants overexpressing the class I barley non-symbiotic hemoglobin gene *HvHb1*. The transgenic lines exhibited improved conversion of NO to NO_3_¯, upregulation of PA biosynthesis genes, and downregulation of ACC synthase as compared to the wildtype (Montilla-Bascón et al. 2017). A similar trend in ET, PAs, and NO was also reported in drought-tolerant transgenic Egyptian cotton varieties but this study also highlighted increased expression of the *SAMDC* gene in transgenic lines as compared to the non-transgenic plants (Momtaz et al. 2010). This enhanced expression of *SAMDC* suggests that SAM during drought stress is diverted for the PA biosynthesis which limits its availability for ET synthesis, leading to reduced ET content. However, a direct inhibitory effect of PAs in ET inhibition has also been shown during drought stress. Exogenous Spm application to the maize plants inhibited ET production under drought stress (Talaat and Shawky 2016). This PA-mediated suppression of ET biosynthesis is likely because of the Spm-mediated inhibition of ACC synthase (Takahashi et al. 2010). Notably, synthesized PAs impart drought tolerance by augmenting membrane stability, scavenging the free radicals to minimize oxidative stress, and ensuring ion homeostasis. These results suggest that drought stress tolerance is predominantly achieved by PAs (Talaat and Shawky 2016). However, some of these PA/Spm-mediated processes such as the maintenance of ROS homeostasis may also require the participation of NO which is known to function downstream of PAs during drought stress (Fig. 5C) (Yamaguchi et al. 2007). Spm-pretreated cucumber seedlings showed early and transient NO production during drought stress that helped the plants adapt to the stress as well as prevent them from subsequent damage (Arasimowicz-Jelonek et al. 2009). Another study showed that NO alone or in combination with PAs can induce drought stress tolerance in sugarcane plants (Silveira et al. 2017) and in white clover where cumulative action of PA (Spd) and NO, generated by the actions of NR and NOS, resulted in enhanced drought tolerance through induction of antioxidant defense system (Peng et al. 2016). In *Rosa damascena*, accumulation of Spd and Spm was observed under moderate and severe drought stress, respectively. This study also revealed that Put catabolism leads to H_2_O_2_ production followed by an increase in NOS expression, potentially leading to NO generation, helping in stomatal closure, and reducing transpiration (Adamipour et al. 2020). Taken together, these results suggest that drought tolerance is majorly achieved by the inhibition of ET biosynthesis and activation of PA synthesis which subsequently promotes NO synthesis and maintenance of ROS homeostasis.

### Iron mineral nutrition

**Fig. 6:**
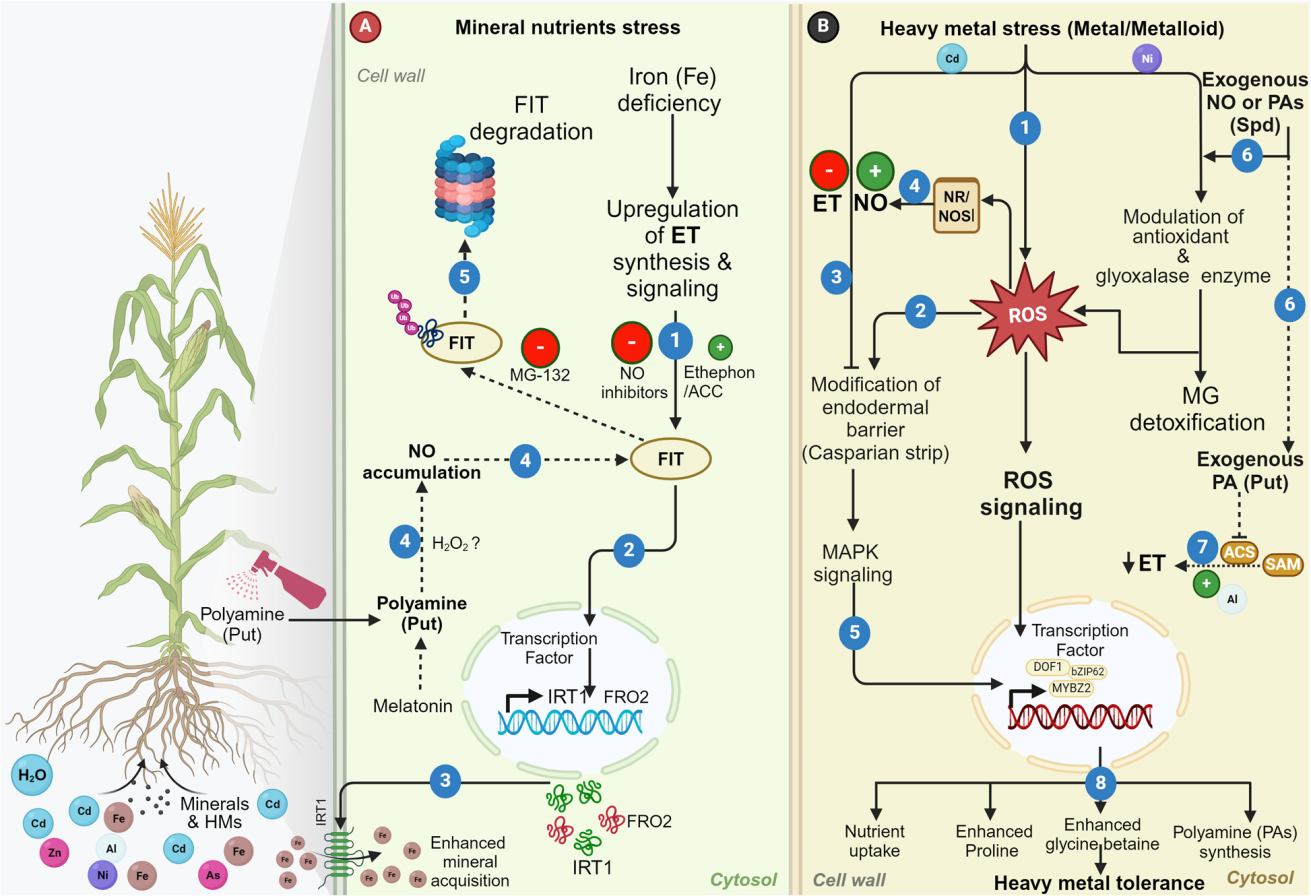
Schematic representation illustrating the interplay of ethylene (ET), nitric oxide (NO), and polyamines (PAs) in response to mineral deficiency and heavy metals (HMs). **A** (1) Under Iron deficiency, Fer-like iron deficiency-induced transcription factor (FIT), is upregulated because of ET biosynthesis. (2–3) FIT leads to the upregulation of key iron acquisition genes such as ferric-chelate reductase (*FRO*) and Iron-regulated transporter 1 1 (*IRT1*), resulting in enhanced mineral acquisition. (4) Exogenous PA (Put) application or melatonin-mediated PA accumulation enhances NO synthesis (probably via H_2_O_2_) that too acts positively on FIT. (5) FIT protein is degraded via proteasome under normal conditions. **B** (1) Plant tolerance to HMs and metalloid toxicity is mediated by different strategies involving ET, NO, and PAs. (2–3) Cadmium (Cd) stress causes induction of ROS that causes the modification of endodermal Casparian strips, however, ET negatively regulates this modification. (4) ROS-stimulated NO generation via NR/NOS, acts positively in the thickening of Casparian strips imparting Cd tolerance to the plant. This suggests an antagonistic interaction between ET and NO during Cd toxicity. (5) The modification of the endodermal Casparian strip involves MAPK-based signaling, inducing TFs such as MYBZ2, bZIP62, and DOF1. (6) Exogenous application of PA or NO as well as Nickel (Ni) stress causes induction of antioxidant synthesis and detoxification of methylglyoxal through the glyoxalase enzyme system. (7) ET level gets downregulated due to elevated PAs during aluminum (Al) stress. (8) Synchronized regulation of HM toxicity by ET, NO and PAs causes gene regulation through key TFs such as bZIP, MYB, and DOF1 that enhance better nutrient acquisition as well as proline, glycine betaine, and PA synthesis

Iron (Fe) is one of the micronutrients required for the proper growth and development of plants (Liang 2022). It is obtained by the plants from their natural reservoir or added as a fertilizer, manure, or amendments (Zelazny and Vert 2014). Experimental results show that ET, NO, and PAs play crucial roles in regulating the absorption and utilization of Fe in plants (Fig. 6A). Plants grown under Fe-deficient soil display upregulation of genes associated with ET biosynthesis and signaling, leading to enhanced ET production (Fig. 6A) (García et al. 2010; Romera et al. 1999). Accordingly, exogenous application of ACC or ethephon mimics morphological growth responses of Fe-deficiency in cucumber and Arabidopsis and enhances expression of genes associated with iron uptake (García et al. 2010; Lucena et al. 2006) (Fig. 6A). In contrast, ET inhibitor treatment abolishes this response suggesting that ET is able to upregulate Fe acquisition involving specific TFs such as FIT-TF that are activated through the ET signaling pathway (Chao et al. 1997; García et al. 2010; Lucena et al. 2006; Romera and Alcantara 1994). FIT is a basic helix-loop-helix TF that is responsible for regulating Fe uptake genes in dicot roots (Meiser et al. 2011). Intriguingly, along with ET, FIT also seems to be regulated by NO as NO inhibitor treatment decreases the FIT protein abundance as well as activity which is reversed by the use of proteasomal inhibitor MG132. These results suggest that the FIT protein is less likely to be targeted for proteasomal degradation in the presence of NO (Fig. 6A) (Meiser et al. 2011). In *A. thaliana,* the exogenous application of Put alleviates Fe deficiency by increasing NO accumulation which helps in mobilizing the cell wall bound Fe (Fig. 6A) (Zhu et al. 2015). Melatonin, a newly recognized PGR, is also reported to trigger PA-induced NO production that subsequently upregulates Fe acquisition genes, *FIT1, FRO2,* and *IRT1* to mobilize cell wall-bound Fe (Zhu et al. 2017) (Fig. 6A). Additionally, NO also increases the soluble iron content and ameliorates leaf chlorosis. These phenomena are completely suppressed in the PA- and NO-deficient plants (Zhou et al. 2016). Therefore, it can be seen that ET, NO, and PAs show a role in Fe nutrition in plants (Fig. 6A).

### Heavy metal stress

Heavy metals (HMs) are relatively high-density elements that exhibit toxic effects by inducing oxidative stress in plants (Riyazuddin et al. 2021). In response to HM toxicity, plants activate signaling cascades that involve NO, ET-PAs synthesis and crosstalk, or NO-PAs interaction (Nahar et al. 2016) (Fig. 6B). A recent report showed that the application of NO can significantly reduce Cd-induced toxicity in rice roots, however, it fails to exhibit any protective effect against As-toxicity (Piacentini et al. 2020). Notably, As-toxicity was effectively mitigated in *Cicer* plants when co-treated with NO and PA (Spd) (Kapoor et al. 2021). The combined presence of NO and PAs modulate the glyoxalase enzyme, leading to significant detoxification of methylglyoxal which alleviates the stress (Kapoor et al. 2021). Likewise, individual as well as combined foliar application of NO and PA (Spm) also alleviate the Nickel (Ni)-toxicity in tomato by activating the glyoxalase enzymes (Gly I and II) and antioxidant defense that triggers proline and glycine betaine accumulation, leading to improved growth, nutrient uptake, pigment synthesis, and photosynthesis rate along with reduced Ni uptake (Qin et al. 2022).

In the case of ET, it has been shown to exhibit antagonistic effects against both NO and PA in the regulation of HM toxicity in plants. For instance, ET displays a negative role while NO exhibits positive effects in the regulation of Cd accumulation in the hyperaccumulator *Sedum alfredii* (Liu et al. 2022) (Fig. 6B). Endodermis, the boundary between cortex and vascular bundle, shows the presence of a unique hydrophobic suberised Casparian strip, a layer that regulates the apoplastic flow of water and uptake of HMs (Liu et al. 2022). It was found that NO positively modifies the formation of the endodermal barrier by promoting a decline in ET concentration during Cd stress (Liu et al. 2022). Application of NO donor resulted in the downregulation of genes involved in ET production which was reversed by NO scavenger cPTIO (Liu et al. 2022). NO donor additionally promoted the deposition of endodermal barriers by upregulating genes associated with suberization whereas high ET level promoted the Cd accumulation and delayed the formation of endodermal barriers (Fig. 6B) (Liu et al. 2021). The crosstalk between ET and PA was revealed in the *T. aestivum* seedlings in response to Aluminium (Al) toxicity. Wheat seedlings display root inhibition during Al toxicity because of the increased ET production at the root apices. However, the application of Put leads to the inhibition of ACS activity which decreases ET production and recovers the Al-induced root inhibition, suggesting an antagonistic role of PAs and ET in Al stress tolerance (Asgher et al. 2018).

In contrast to the above reports that suggest a negative role of ET, some reports also show its protective effects in HM stress mitigation. For instance, ET treatment reduced the oxidative stress induced by Ni and Zn in mustard (*Brassica juncea*) by inducing antioxidant machinery along with ensuring efficient PSII and RUBISCO activities and improved photosynthetic nitrogen use efficiency (Fig. 6B) (Khan and Khan 2014). MAPKs are involved in the regulation of ET biosynthesis and signaling (Guo and Ecker 2004; Hahn and Harter 2009). It was found that Cd tolerance leads to the activation of MAPK6, mediated by NO, suggesting it to be a connecting link between NO and ET signaling (Thao et al. 2015; Ye et al. 2013). These results can be further corroborated by another study which showed that short-term Cd stress induces ET, PA, NO, MAPKK2, and TFs such as MYBZ2, bZIP62, and DOF1 in young soybean seedlings (Fig. 6B). Bioinformatic analysis revealed that several regulatory motifs present in the promoter sequences of Cd-induced genes are associated with the ET signaling which further suggests a crucial role of ET in the induction of those genes (Chmielewska-Bak et al. 2014).

## Conclusion and future perspectives

ET and NO are both small gaseous compounds. However, they are significantly different in their origin, chemical nature, and signaling pathways. ET, NO, and PAs are generated from interconnected biological pathways and hence generation of one affects the biosynthesis and signaling of the others. Among these, the exact role of ET in stress tolerance is enigmatic and seems to be stress and plant-dependent. The common substrate SAM seems to be utilized for the synthesis of ET only during the early stages of stress conditions while it is preferentially utilized for the synthesis of PAs during the later stages. The activation of ET signaling at the onset of stress conditions possibly prepares plants to tolerate the stress while PA biosynthesis and their oxidation in the later stages generate NO and H_2_O_2_ that again activate downstream signaling to alleviate stress conditions. NO can also be formed at the early stages of stress, independent from PA oxidation, by the activity of NOS-like or NR enzyme activities which exhibits stress mitigatory effects. NO, in turn, regulates ET biosynthesis, thereby forming a feedback loop. NO, if formed during the initial stages of stress, triggers ET biosynthesis by inducing S-nitrosylation of ET biosynthesis proteins while suppressing its own synthesis to ensure the attenuation of ET biosynthesis/signaling during later stages of stress by forming NO-ACC-ACO ternary complex. Notably, the crosstalk of these molecules is discussed mostly in a pairwise manner such as ET-NO, ET-PA, or NO-PA and there is a paucity of reports discussing the trio together. The lack of availability of such data makes it interesting to connect these molecules in a comprehensive manner for a better understanding of their effects and responses in overcoming environmental stresses.

## Data Availability

No data was generated for this manuscript.

## Abbreviations

ACC: 1-Aminocyclopropane-1-carboxylic acid
ACS: ACC synthase
ADC: Arginine decarboxylase
AOX: Alternative oxidase
CDPK: Calcium-dependent protein kinase
DAO: Diamine oxidase
ET: Ethylene
HM: Heavy metal
HSP: Heat shock proteins
NO: Nitric oxide
NOS: Nitric oxide synthase
NR: Nitrate reductase
ODC: Ornithine decarboxylase
PA: Polyamine
PGR: Plant growth regulator
Put: Putrescine
ROS: Reactive oxygen species
SAM: S-Adenosyl methionine
SAMDC: S-adenosylmethionine decarboxylase
SAMS: Spermine synthase
Spd: Spermidine
SPDS: Spermidine synthase
Spm: Spermine
UV: Ultraviolet
